# A Comparative Study on Knowledge about Reproductive Health among Urban and Rural Women of Bangladesh

**Published:** 2015-03

**Authors:** Monoarul Haque, Sharmin Hossain, Kazi Rumana Ahmed, Taslima Sultana, Hasina Akhter Chowdhury, Jesmin Akter

**Affiliations:** 1 Department of Community Nutrition, Faculty of Public Health, Bangladesh University of Health Sciences (BUHS) Dhaka, Bangladesh; 2 Department of Health Promotion & Health Education, Bangladesh University of Health Science (BUHS), Dhaka, Bangladesh; 3 Department of Public Health, State University of Bangladesh, Dhaka, Bangladesh; 4 Department of Biostatistics, Bangladesh University of Health Sciences (BUHS), Dhaka, Bangladesh; 5 Department of Reproductive and Child Health, Bangladesh University of Health Sciences (BUHS), Dhaka, Bangladesh

**Keywords:** Knowledge, Reproductive Health

## Abstract

**Objective:** To compare the level of knowledge on reproductive health among urban and rural women of selected area of Bangladesh.

**Materials and methods:** A descriptive cross-sectional study was undertaken among 200 women selected purposively from different rural and urban areas of Bangladesh. Data were collected using a semi-structured interviewer-administered questionnaire by face to face interview. Knowledge level was analyzed according to poor, moderate and good knowledge by pre-defined knowledge scoring.

**Results:** Mean age of the respondents was 26 years and majority (66%) of them was housewives. Most of them (61%) had completed their primary level education. Around three-fourth of them belongs to lower-middle income group. Overall level of reproductive health knowledge was more evident among urban reproductive aged women than rural counterparts (p < 0.001). Moreover, significant knowledge gap was found regarding family planning (p = 0.005), care during pregnancy (p < 0.001), safe motherhood (p = 0.002), newborn care (p = 0.009) and birth spacing (p <0.001) between urban and rural women. Family members were the major source of information in both groups.

**Conclusion:** A wide knowledge gap was found between Bangladeshi urban and rural respondents regarding their reproductive behaviors. Government and concerned organizations should promote and strengthen various health education programs to focus on reproductive health, especially among reproductive aged women in rural area.

## Introduction

Reproductive health has been a great concern for every woman. It is a crucial part of general health and a central feature of human development. Reproductive ill-health have been a apprehension to many stakeholders as maternal mortality and morbidity are very high in developing countries, especially in Bangladesh compared to developed world. In the past few years, the issues of Reproductive Health/Rights (RH/RR) have been increasingly perceived as social problems; they have emerged as a matter of increasing concern throughout the developed and developing countries. Bangladesh has achieved remarkable progress in important aspects of health and family welfare since Independence. However, the overall health status, particularly the status of reproductive health, still remains unsatisfactory (1). The insufficient health services available to women and children are evident from high infant and maternal mortality rates (2, 3). 

The common health problems faced by both rural and urban women of Bangladesh are lower abdominal pain accompanied by heavy bleeding, white discharge and irregularity of the menstrual cycle (4, 5). The major concern, though, is that they do not discuss these since they do not consider these normal illnesses. Although urban educated women sometimes visit doctors, but women in rural areas are taken to some traditional healers, like Kabiraj/Hakim, who prescribes Tabij and herbal medicines, Pir (saints), Fakir (religious persons) or Huzurs (mullah), who prescribes Panipora (sanctiﬁed water) (5). Family planning helps women to protect from unwanted pregnancies, thereby saving them from high risk pregnancies or unsafe abortions. 

Health knowledge is considered as one of the key factors that enable women to be aware of their rights and health status in order to seek appropriate health services. It is very important to study the overall situation and to know the differences between rural and urban Bangladeshi women in order to focus on reproductive health issues. The results could be used as an important guide to assist policymakers and administrators in evaluating and designing the programs and strategies for improving reproductive health services with a special consideration for rural women. 

Modern facilities, such as TV, radio, newspaper, etc., have played a vital role to ensure the reproductive health of the respondents. Possession of modern facilities is a very important issue for a society, and a society with enough modern facilities is more developed, while people enjoy their reproductive health. Consequently, mass media such as radio and television can create awareness about issues affecting the daily life, family planning programs, poverty alleviation programs, gender issues, human rights issues, etc. 

The focus of this study, therefore, was to find out the level of knowledge regarding reproductive health among rural and urban women of Bangladesh.

## Materials and methods

A cross sectional study was conducted among 200 reproductive age group women (15-49 years), whereas 100 respondents were randomly selected from urban community of Dhaka and rest of 100 were selected from rural community of Sirajgonj. This area was selected purposively to get adequate sample. The study subjects were included women who sought reproductive health services in the health facilities like antenatal care, self immunization during pregnancy, child immunization, choice of family planning methods and safe abortions, and who were willing to participate and to provide required information. Physical and mental retarded people and very sick were excluded from the study. The study consisted of both data gathered by structured and semi structured questionnaires and in depth interviews with Bangladeshi women. The field work was conducted from April to July 2013 in four clinics/hospitals providing reproductive health care for women from the two districts: Dhaka and Sirajgonj. Out of the four hospitals, two were selected from urban areas in Dhaka City Corporation (Marie Stopes Clinic and Dhaka Medical College Hospital, Dhaka), and other two hospitals were selected from rural community of Sirajgonj (Kamarkhando and Belkuchi Upazilla Health Complex, Sirajgonj), which was about eighty kilometers away from Dhaka. The socioeconomic scenario of rural areas of Bangladesh were almost same all over the country with lack of roads, transportations, sanitary facilities, electric supply, women education, and women empowerment, while they have less access to mass media, newspapers, health service facilities and so forth. The clinics/hospitals were selected purposively, and from one clinic/hospital, fifty respondents were interviewed. The respondents were selected consecutively who meet the inclusion and exclusion criteria. Data were collected by interviewer-administered questionnaires. The socio-economic classification in this study was made according to 2006 Gross National Income (GNI) per capita using the calculation of World Bank (WB) (6) [The groups were: low-income $75.41 or less (BDT ≤ 5360), lower middle-income $75.5 - $299.58 (BDT 5361-21270), upper middle-income $299.68 - $926.25 (BDT 21271-65761) and high-income $926.33 or more (BDT ≥ 65762)]. It is noteworthy that scores 0 and 1 were allocated to each of the questions of reproductive knowledge. Three categories were defined on the basis of the score obtained by each participant: poor (<50% of the total score), moderate (50%-70% of the total score), and good (>70% of the total score). After collection, data were checked thoroughly for consistency and completeness, and all analysis was done by appropriate statistical methods using Statistical Package for Social Sciences (SPSS**; **SPSS Inc., Chicago, IL, USA) software for Windows version 16.0. Univariate and bivariate analysis were done as appropriate to show results.

## Results

The study results revealed that among the 200 respondents, mean (± SD) age of them was 26±5 years. Among the respondents, 50% belonged to urban and another 50% were from rural areas. Most of them (61%) had primary education; this was followed by those who (9%) had higher secondary level of education. Preponderance of housewives (66%) was observed, whereas rest of them was involved with NGO job, garments work and student. Majority of the respondents belonged to lower middle income family (77%), and only 1% was from high income family (Table 1).

**Figure 1:**
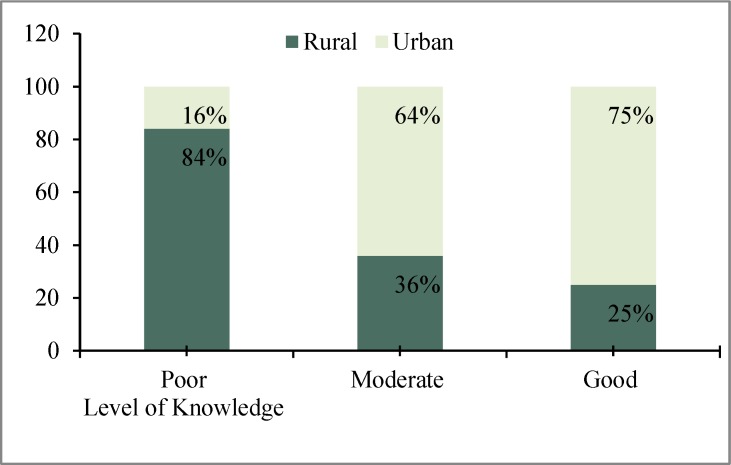
Overall Level of Knowledge on reproductive

The figure shows the proportion of poor knowledge regarding reproductive health was more (84%) among rural women, but good knowledge was more rampant (75%) among urban women. The overall knowledge difference between urban and rural women regarding reproductive health were highly significant (p < 0.001) (Figure 1).

The extent of respondent’s knowledge on different components of reproductive health showed that rural women had less knowledge on family planning than urban women. Family planning refers to deliberate efforts of couples or individuals to regulate fertility by delaying or spacing births or limiting the number of their children. Significant association was found between location of respondents and knowledge on family planning (p = 0.005). Knowledge regarding contraceptive method use reveals that everybody had better knowledge on contraceptive use. Around 88% rural women had moderate knowledge on contraception. But in terms of good knowledge, urban women went ahead than rural women. Overall Level of Knowledge score did not differ between groups (p = 0.542) (Table 2).

**Table 1:** Socio-demographic information of the respondents (n = 200)

Parameters	n (%)
**Age (M ± SD) **	**26 ± 5**
**Religion**	
** Muslim**	**184 (92)**
** Hindu**	**16 (8)**
**Education of respondents**	
** No schooling**	**9 (5)**
** Primary**	**122 (61)**
** Secondary**	**52 (26)**
** Higher secondary**	**17 (9)**
**Occupation of respondents**	
** Housewife**	**133 (66)**
** NGO job**	**21 (10)**
** Teacher**	**17 (9)**
** Garments worker**	**15 (8)**
** Student**	**14 (7)**
**Monthly income (BDT)**	
** Lower income (< 5360)**	**28 (14)**
** Lower middle income (5360-21270)**	**154 (77)**
** Upper middle income (21271-65761)**	**16 (8)**
** High income (> 65761)**	**2 (1)**

Results are expressed as number (%) and Mean ±SD

health among urban and rural women (χ2 = 41.737; p < 0.001)

Moreover, table 2 indicates that urban women had (56%) good knowledge on care during pregnancy. But in case of safe motherhood, both urban (96%) and rural (83%) women had good knowledge (p = 0.002). Urban women (40%) had good knowledge on new born care where 18% rural women had no knowledge regarding it, and the difference was significant (p = 0.009). Around 72% and 70% urban and rural women had good knowledge on safe abortion, respectively. However, it is cheering that a total of 90% urban women had good knowledge on birth spacing and family size, but around one-third (36%) of rural women did not have any knowledge on birth spacing and family size (Table 2).

**Figure 2:**
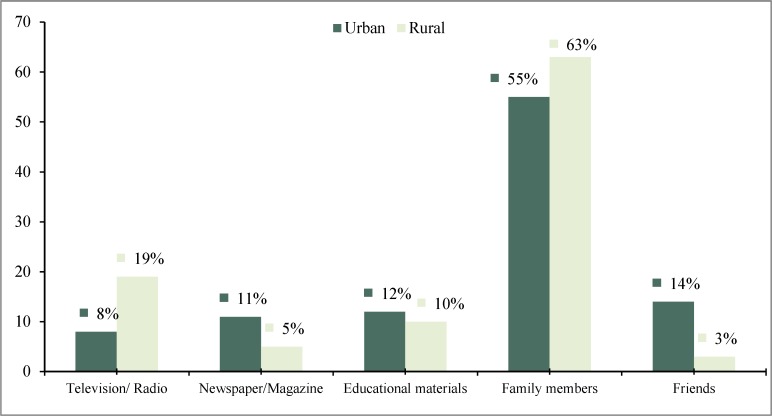
Source of information on reproductive health among urban and rural women

In respect to the source of information regarding reproductive health and reproductive rights, 55% urban and 63% rural women revealed that the most important source of information was their family members such as their spouse, parents, siblings, cousins, etc. In urban area, only 8% women gathered information from television or radio, while the corresponding value for rural women was 19%. The responds also congregated information from newspaper or magazine (11% vs. 5%), other educational materials (12% vs. 10%) and friends or peers (14% vs. 3%), respectively (Figure 2).

**Table 2:** Level of Knowledge on different components of reproductive health among urban and rural women (n = 200)

Level of knowledge	Urban	Rural	p value
**Knowledge on family planning**			
** Poor**	**26**	**42**	**0.005**
** Moderate**	**6**	**0**	
** Good**	**68**	**58**	
**Knowledge on contraceptive method use**			
** Poor**	**0**	**0**	**0.542**
** Moderate**	**84**	**88**	
** Good**	**16**	**12**	
**Knowledge on care during pregnancy**			
** Poor**	**30**	**56**	**0.001**
** Moderate**	**14**	**0**	
** Good**	**56**	**44**	
**Knowledge on safe motherhood**			
** Poor**	**2**	**17**	**0.002**
** Moderate**	**2**	**0**	
** Good**	**96**	**83**	
**Knowledge on newborn care**			
** Poor**	**0**	**18**	**0.009**
** Moderate**	**60**	**50**	
** Good**	**40**	**32**	
**Knowledge on safe abortion**			
** Poor**	**28**	**30**	**0.876**
** Moderate**	**0**	**0**	
** Good**	**72**	**70**	
**Knowledge on birth spacing and family size**			
** Poor**	**10**	**36**	**0.001**
** Moderate**	**0**	**0**	
** Good**	**90**	**64**	

Results were expressed as frequency percentage; χ2 test was performed and p < 0.05 was level of significance.

(*multiple responses)

## Discussion

Reproductive health knowledge is important for women as woman's health and well-being, contraception, as well as those of her family may depend on her being able to delay the birth of her first child or space the birth of her children (7, 8). 

Women started antenatal care at a relatively early stage of their pregnancy (before 4-month pregnancy), and 78.2% of women made six or more antenatal care visits during their entire pregnancy, but present study found that most of the respondents made three or four antenatal care visits during their entire pregnancy which was not similar to Palestine study (9). On the other hand, our study found that poor knowledge on reproductive health was more among rural women (84%). Urban women were more knowledgeable about reproductive health than rural women. Thus overall knowledge on reproductive health among urban women was better than rural women.

Women are often aware of benefits of family planning. Women's decision about use, non-use, or discontinuation of family planning methods can be affected by their perceptions of contraceptive risks and benefits, concerns about how side effects may influence their daily lives and assessment of how particular methods may affect relationships with partners or other family members (10, 11). A study was done to understand the family planning (FP) knowledge and current use of contraception and its predictors among women of the Mru people – the most underprivileged indigenous community in Bangladesh (12). Only about 40% of respondents had ever heard FP messages or about FP methods – two-fifths of the national figure (99.9%). The current use of contraception was much lower (25.1%) among the Mru people than at the national level (55.8%) (12). But our study found that 58% rural women had good knowledge on family planning, whereas 68% urban women showed good knowledge on family planning.

Safe motherhood has evolved from a neglected component in maternal and child health programs to an essential and integrated element of women’s sexual and reproductive health. A comparative case study was done in Nepal on safe motherhood practice and they showed that more than a half have knowledge about safe motherhood, but practice is found lower as compared to the magnitude of known. Antenatal care is found better in comparison about safe motherhood which is found some increased now than before. Family planning knowledge is found better as compared to antenatal, safe delivery and postnatal cares among this caste (13). Present study found that most of the study subjects both urban and rural had good knowledge on safe motherhood.

Unsafe abortions still contribute to 13-50 % of the maternal mortality in some of these countries. Only three respondents have actually dealt with abortion related cases. A few respondents said that they have dealt with issues like adultery, extra marital relations, etc. but not with abortions. The source of their information on abortion was, therefore, media or other reports and TV as like my study subjects. However, seventeen of them were interested in knowing more about abortion and abortion laws (14).

A study aimed at identifying the effect of birth spacing on maternal health among 324 married women at the fertile age period and revealed that women practicing birth spacing were 33.3% of the studied sample. Good knowledge and favorable attitude were 88% and 100% of properly birth spaced women, respectively. Rural origin women were less practicing birth spacing (15). The present study found that 90% urban women had good knowledge on birth spacing and family size, but 64% rural women had knowledge on birth spacing and family size.

On the basis of this study, knowledge on reproductive rights was more pronounced among urban reproductive women than rural. The present study found that family planning and contraceptive knowledge factors were less among rural reproductive women. Out of 200 study subjects, nearly half had knowledge about care during pregnancy, but knowledge on safe motherhood was satisfactory in both urban and rural area. Knowledge on safe abortion is found less good in comparison about safe motherhood. Besides 90% urban women had good knowledge on birth spacing and family size, but 64% rural women had knowledge on birth spacing and family size. 

## Conclusion

A wide gap was found between urban and rural respondents in Bangladesh regarding their reproductive behaviors and exercising their reproductive rights.

## References

[1] Hasan MK (2005). Reproductive rights and decision-making: A comparative study in rural and urban Bangladesh. Perspectives in Social Sciences.

[2] Global Policy Committee of the World Health Organization 2 May 1994; and the WHO Position paper on health, Population and Development, Cairo 5-13.

[3] The center for Reproductive Rights (2005). Women of the world, South Asia.

[4] Khan MR (1997). Report on Focus Group Discussion on Maternal Health, Report prepared for UNICEF.

[5] Huq N, Khan M (1991). Menstruation: Beliefs and Practices of Adolescent Girls.

[6] Haque ANMN (2007). The middle-income matrix. The Daily Star.

[7] Smith EJ (2002). Protecting fertility Network.

[8] Nazar-Beutelspacher A, Matina-Rosales D, Salvatierra-Ilzaba B, Zapata-Martelo E, Halperrin D (1999). Education and non-use of contraceptives among poor women in Chiapas, Mexico. Int Fam Plann Perspect.

[9] Dhaher E (2008). Reproductive health human rights: women's knowledge, attitude, and practices toward their reproductive health rights in Palestine.

[10] Marchant T, Mushi A K, Nathan R, Mukasa O, Abdulla S, Lengeler C (2004). Planning a family: Priorities and concerns in rural Tanzania. Afr J Reprod Health.

[11] Ndong I, Finger WR (1998). Male responsibility for reproductive health. Introduction. Netw Res Triangle Park N C.

[12] Islam MR, Thorvaldsen G (2012). Family planning knowledge and current use of contraception among the Mru indigenous women in Bangladesh: a multivariate analysis. Open Access Journal of Contraception.

[13] Oli K (September 2007). Safe motherhood practices in Rukum.

[14] Rahman A, Katzive L, Henshaw SK (1998). A global review of laws on induced abortion, 1985-1997. Int Fam Plann Persp.

[15] el-Maaddawi Y, Aboul-Ella MN, Abdel-Moneim MN, Tantawy AB (1992). Study of the effect of birth spacing on maternal health. J Egypt Public Health Assoc.

